# Kinetics of the O_2_ Reactions with CH_3_OCO and CH_2_OCHO Formed from Methyl Formate

**DOI:** 10.1021/acs.jpca.6c01296

**Published:** 2026-04-16

**Authors:** Lavinia Onel, Robin Shannon, Niamh C. K. Robertson, Mark A. Blitz, Daniel Stone, Paul W. Seakins

**Affiliations:** † School of Chemistry, 4468 University of Leeds, Leeds LS2 9JT, U.K.; ‡ National Centre for Atmospheric Science, University of Leeds, Leeds LS2 9JT, U.K.

## Abstract

Methyl formate (CH_3_OCHO, MF) is the simplest ester, a class of species that is of interest for combustion and atmospheric chemistry studies. H-abstraction reactions from the two sites of MF leads to radicals, R, that react with oxygen to form RO_2_ species that are important in the oxidation of MF under atmospheric and low temperature combustion conditions. This work reports the kinetics of the O_2_ reactions with R formed through the reaction of Cl with MF from 213 K to ∼420 K and in the range ∼5–100 Torr of N_2_ or Ar. The rate coefficients *k*_R+O_2__ were determined by the analysis of the kinetic profiles of the OH radicals formed by the prompt dissociation of RO_2_, which were measured using the pulsed laser flash photolysis–laser induced fluorescence technique. At 294 K in N_2_ (5–100 Torr), *k*_R+O_2__ was pressure independent, within experimental error, with a value of (5.0 ± 0.4) × 10^–12^ cm^3^ molecule^–1^ s^–1^. Isotopic studies using CH_3_OCDO were used to differentiate between the kinetics of the CH_3_OCO and CH_2_OCHO associations with O_2_. CH_2_OCDO reacted faster than CH_3_OCO by approximately 40% at 213 K, rising to 200% at 472 K. The enhanced reactivity of the CH_2_OCHO with O_2_ can be explained by a more attractive potential energy surface and a looser transition state. It was also possible to determine the rate coefficients of the unimolecular decomposition of CH_3_OCO, *k*_dec_ (348–470 K). The experimental values of *k*_dec_ were fitted with the master equation application, MESMER, with the literature (ANL0F″) barrier height of 57.3 kJ mol^–1^, and floating the average energy transfer parameter at 298 K, <Δ*E*>_d, 298 K_, and the temperature exponent of <Δ*E*>_d_. A good fit to the data was obtained with: <Δ*E*>_d_(Ar) = (110 ± 30) × (*T*/298 K)^(0.0 ± 0.7)^ cm^–1^ and <Δ*E*>_d_(He) = (34.4 ± 6.2) × (*T*/298 K)^(1.0 ± 0.5)^ cm^–1^.

## Graphical abstract

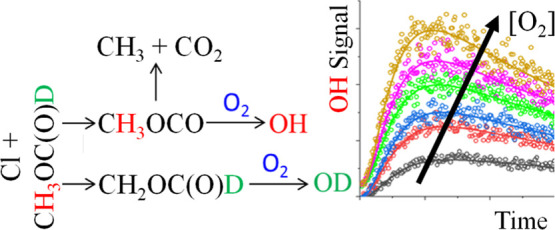

## 1 Introduction

Esters are a component of biodiesel, which is a renewable, reduced carbon fuel and a viable alternative for conventional fossil diesel. Typically, biodiesel is composed of fatty acid methyl esters (FAMEs) and fatty acid ethyl esters (FAEEs).^1,2^ Biodiesel is mostly used in blends with petroleum diesel and has a range of beneficial effects on engine emissions owing to reduced CO, hydrocarbon and particulate matter emissions.^3^ Additional advantages of the use of biodiesel blends include the rise in the cetane number and an improvement in fuel lubricity.^4^

The complexity of the molecular structure of the fatty acids esters, which involve large aliphatic chains, poses a challenge to modeling and experimental kinetic studies. As methyl formate (MF) is the smallest methyl ester and thus the simplest surrogate in the FAMEs family, MF has been chosen as a model compound to study the combustion chemistry of methyl esters.^5,6^ In addition, MF is an intermediate in the low-temperature combustion (LTC) of dimethyl ether^7^ and dimethoxymethane,^8^ which are also promising diesel alternatives.

MF is emitted into the Earth’s atmosphere from vegetation and anthropogenic sources such as solvents and in the manufacturing of perfumes and flavorings. Methyl formate is also formed as an oxidation product of methyl-ethers in the atmosphere.^9–11^ The atmospheric oxidation of MF is typically initiated through H-abstraction by OH radicals, which produces radicals, R,; CH_3_OCO following abstraction at the formate site and CH_2_OCHO following abstraction at the methyl site.^12^ Under atmospheric conditions, these R species associate with O_2_ to generate peroxy radicals (RO_2_)^13^

R1 $$C_{2}H_{3}O_{2}+O_{2}\overset{k_{1,R+O_{2}}}{\to }Prod$$

R1a $${CH}_{3}OCO+O_{2}\overset{k_{1a,{CH}_{3}OCO+O_{2}}}{\to }{CH}_{3}{OC\left(O\right)O}_{2}$$

R1b $${CH}_{2}OCHO+O_{2}\overset{k_{1b,{CH}_{2}OCHO+O_{2}}}{\to }O_{2}{CH}_{2}OCHO$$

where C_2_H_3_O_2_ = RO_2_, the combination of both isomers.

The LTC of biodiesel has attracted recent interest;^14,15^ fuel consumption in LTC is typically initiated by reaction with OH, resulting in the generation of radicals, R, which then react with O_2_ to produce RO_2_. RO_2_ species can undergo internal rearrangement to form a carbon-centered QOOH radical.^16^ The autoignition of the fuel is believed to occur through the sequence of reactions: QOOH + O_2_ → O_2_QOOH → OH + Q′OOH → chain branching. The O_2_ addition to QOOH is in competition with the QOOH decomposition in a chain propagating reaction: QOOH → OH + carbonyl. QOOH + O_2_ reactions are difficult to isolate for study; more data on R + O_2_ reactions, particularly involving oxygenated R species, would enhance our ability to realistically estimate QOOH + O_2_ kinetics.

Under LTC conditions, the reaction of R with O_2_ can also be in competition with the unimolecular decomposition of R.^17,18^ A number of theory studies^19–21^ have been carried out to study the decomposition of CH_3_OCO formed by the H-abstraction from the formate-site of MF and the oxidation of larger FAMEs.^22,23^ It was found that the main channel of the CH_3_OCO decomposition is a β-scission leading to CH_3_ and CO_2_ (R2).^19–21^

R2 $${CH}_{3}OCO\overset{k_{2,dec}}{\to }{CH}_{3}+{CO}_{2}$$

Reaction R2 is expected to dominate the fate of CH_3_OCO under LTC conditions. Because of the relatively weak H_3_C–O bond, the potential of CH_3_OCO to undergo prompt decomposition through a “hot β-scission” ^24,25^ has been experimentally and theoretically investigated.^12^ No evidence for the process occurrence at room temperature was found, showing that the addition of O_2_ is the dominant reaction of CH_3_OCO at room temperature,^12^ in contrast to the Master Chemical Mechanism (MCM)^26^ that assumes 100% decomposition of CH_3_OCO under atmospheric conditions.

Due to the exothermicity of the R + O_2_ reactions, the RO_2_ species are formed with excess energy. In laboratory experiments, the conditions can be arranged such that chemical activation (CA), leading to OH regeneration through the decomposition of the highly energetic RO_2_ (RO_2_*) occurs. CA is promoted at low pressures, where the collisional stabilization of the highly energetic species RO_2_* is less important.
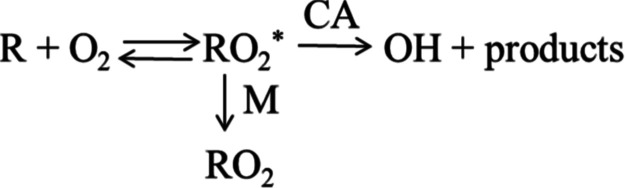


Details on the production of OH from RO_2_ and RO_2_* will be presented in a future paper focusing on RO_2_ to QOOH isomerization and QOOH decomposition to OH.

Despite their significance under LTC conditions (particularly the competition between R + O_2_ and R decomposition) and in the Earth’s atmosphere, the kinetics of the R + O_2_ reactions from MF are, to the best of our knowledge, unknown, although OH production from the Cl/MF/O_2_ system has been observed.^27^ This is the first study of the rate coefficients *k*_R+O_2__ for the MF system. In the present work, R species were generated through the reactions of Cl with MF (R3). The reactions with Cl were chosen as they are significantly faster than the OH reactions with MF (∼ factor 10 at 298 K).^12,28^ Reaction R3 occurs through two channels, i.e. the H-abstractions from the ester-group (R3a) and the methyl group (R3b).

R3a $$Cl+{CH}_{3}OCHO\overset{k_{3a,Cl+MF}}{\to }{CH}_{3}OCO+HCl$$

R3b $$Cl+{CH}_{3}OCHO\overset{k_{3b,Cl+MF}}{\to }{CH}_{2}OCHO+HCl$$

In this work the kinetics of the R + O_2_ reactions were studied by measuring the temporal profiles of the OH radicals formed by the RO_2_* species using the laser flash photolysis–laser induced fluorescence (LFP–LIF) technique (Scheme 1).

**Scheme 1:**
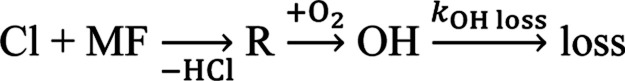


The measured OH vs time profiles in the range 213–420 K were analyzed by fitting to the triexponential eq E1, which describes the reaction system shown in Scheme 1.

E1 $$\left[OH\right]=A\cdot \left[B_{1}\exp\left(-C_{1}t\right)+B_{2}\exp\left(-C_{2}t\right)+B_{3}\exp\left(-C_{3}t\right)\right]$$

The terms of the eq E1, A, B1–B3 and C1–C3 depend on the rate coefficients of the reactions included in Scheme 1: *k*_3,Cl+MF_, *k*_1,R+O_2__ and *k*′_OH loss_, i.e. the bimolecular rate coefficients for the Cl + MF and R + O_2_ reactions and the rate coefficient describing the OH loss by reaction with MF and diffusion out of the observation volume (Results and Discussion section). For a robust analysis, the bimolecular rate coefficient *k*_3,Cl+MF_ was constrained to the values determined in separate experiments using sufficiently large concentrations of O_2_ to ensure that the R + O_2_ reactions were not rate-determining. Under such conditions the Cl + MF reaction is the rate-determining step in the formation of OH, thus the triexponential eq E1 reduces to a biexponential one (equation ES1 in Supporting Information, Section S1).

The Cl + MF experiments did not allow us to discriminate between *k*_1,R+O_2__ for the two types of radicals generated by reactions R3, i.e. CH_3_OCO or CH_2_OCHO, as both radicals lead to OH through reaction with O_2_. To differentiate between *k*_1a,CH_3_OCO+O_2__ and *k*_1b,CH_2_OCHO+O_2__, experiments using the Cl reaction with deuterated MF, CH_3_OCDO (MF-d1) were performed to measure OH and OD formed with O_2_. In addition, the deuterated esters experiments enabled the determination of the rate coefficients for the unimolecular decompositions of CH_3_OCO, *k*_2,dec_, in the temperature range 348–470 K. Analysis of experimental results, using the master equation solver for multi-energy well reactions (MESMER),^29^ were performed to study the temperature dependence of *k*_2,dec_ in Ar or He at pressures between 10–50 Torr.

Overall rate coefficients for the Cl + MF and Cl + MF-d1 reactions were also determined. The present measurements of *k*_3,Cl+MF_ were performed from 213 to 420 K and thus extend the temperature range of the previous experimental studies of the kinetics of the Cl reactions with MF.^28,30–33^

This paper is one of a series of publications on ester oxidation. In Robertson et al.,^12^ we examined the site-specific abstractions of OH radicals with methyl formate. In this paper, we look at the kinetics of the resultant radicals with oxygen (R + O_2_ → RO_2_) and their decomposition (R → products). In a future paper, we will report on the RO_2_ isomerization to QOOH and generation of OH from both chemically activated and thermal reactions of RO_2_.

## 2 Experimental

The experiments were performed in a conventional slow–flow pulsed laser flash photolysis–laser induced fluorescence apparatus.^34,35^ For the experiments carried out above room temperature, the reaction cell was heated using a series of cartridge resistance heaters surrounding the cell, with temperatures monitored by a K-type thermocouple situated close to the reaction zone as described previously.^34^ For the investigations at subambient temperatures a second reaction cell, which was immersed in an insulated metal bath,^36^ was incorporated in the apparatus. The bath was filled with a thermofluid (Huber DW-Therm) and surrounded by 3 cm thick polystyrene for insulation. The temperature for this cell was controlled employing a refrigerated immersion chiller (LabPlant RP-100CD) with the probe immersed in the thermofluid and a paddle stirrer used to achieve temperature uniformity.

MF (Sigma-Aldrich, 99% purity) and CH_3_OC­(O)­D (MF-d1, Cambridge Isotope Laboratories, 99.9% D atom) were stored as dilute mixtures (in nitrogen, helium or argon) in glass bulbs. The OH precursor, ester (MF, MF-d1), O_2_ (BOC, 99.999%), and the bath gas—N_2_ (BOC, oxygen free, 99.99%), He (BOC, 99.99%) or Ar (BOC, 99.99%)—were introduced through calibrated mass flow controllers into a mixing manifold and flowed through either the heated or the cooled reactor. The reactions were studied under pseudo-first order conditions, using concentrations of ester in the range 10^14^–10^15^ molecule cm^–3^. The concentration of oxygen depended on the type of experiment: [O_2_] ∼10^14^–10^15^ molecule cm^–3^ in the experiments performed to obtain the rate coefficients for the R + O_2_ reactions and [O_2_] ∼10^16^–10^17^ molecule cm^–3^ in the experiments carried out to measure the rate coefficients for the Cl + MF reactions. The total pressure in the reaction cells was regulated by throttling the exit valve and measured using a capacitance manometer.

The Cl precursor was oxalyl-chloride, (COCl)_2_ (>99%)^37^

R4 $${\left(COCl\right)}_{2}+h\nu \backslash \left(\lambda =266nm\right)\to CO+Cl+COCl/CO+Cl$$

The OH precursor was H_2_O_2_ (50% v/v aqueous)

R5 $$H_{2}O_{2}+h\nu \left(\lambda =266nm\right)\to 2OH$$

To entrain the OH precursor into the gas flowing toward the reactor, N_2_ carrier gas of a known flow rate was passed through a bubbler containing degassed H_2_O_2_. Both the Cl and OH precursors were photolyzed at 266 nm using an Nd:YAG laser (Quantel Q-smart 850), at a typical laser fluence of 50 mJ cm^–2^ pulse^–1^ and repetition frequency of 10 Hz.

OH and OD were probed by on-resonance laser induced fluorescence following excitation at around 308 nm corresponding to the A^2^Σ­(ν′ = 0) ← X^2^Π­(ν″ = 0) transition (∼307.248 and ∼307.285 for OH and OD). The probe laser light was generated using the 532 nm output of an Nd:YAG (Continuum Powerlite 8010) to pump a dye laser (Sirah Cobra Stretch, 10 Hz) operating on DCM Special dye.

Time-dependent profiles of OH/OD fluorescence signal were built up by varying the delay time between the photolysis and the probe pulses. Kinetic profiles were averaged for between 5 and 10 traces depending on the signal quality. The reactions were carried out under pseudo-first order conditions with [ester] ≫ [Cl]_0_ in the experiments using Cl and [MF] ≫ [OH]_0_ in the OH + MF/O_2_ system (Supporting Information, Section S2). OH/OD fluorescence–time profiles are shown in the Results and Discussion section.

## 3 Calculations

Calculating quantitative rate coefficients for barrierless reactions is challenging, requiring variational transition state theory and by extension, a large number of ab initio calculations mapping the conformational space as the two reacting moieties approach. This is complicated by the fact that as a radical and neutral species approach, the kinetic bottleneck is generally in regions where the electronic wave function is poorly described by a single determinant requiring multireference calculations to be accurately described.^38–40^ Given the experimental focus of this work, we have taken a qualitative approach to the calculations. We have calculated relaxed scans for the reactions of both radicals R with O_2_, with the distance between the radical site on the R and one of the oxygen atoms varied from 4.5 to 1.5 Å. These calculations were performed with the rs2 variant of CASPT2 as implemented in Molpro^41^ with a (5o, 7e) active space including the oxygen Π orbitals and the radical center. An aug-cc-pVDZ basis set was used for these calculations.

We have modeled the R + O_2_ experimental rate coefficients using master equation simulations in MESMER.^29^ The barrierless association reactions were treated with an inverse Laplace transform (ILT) method and high-pressure limiting rate coefficient *k* ^∞^(*T*) of the form

E2 $$k^{\infty }\left(T\right)=A{\left(\frac{T\left(K\right)}{298K}\right)}^{n}$$

The *A* and *n* parameters were optimized through a Levenberg–Marquardt fitting routine within MESMER.

To model the fall off behavior, energy transfer was modeled using an exponential down model with the average energy transfer upon a downward collision (⟨Δ*E*⟩_d_) fit to the experimental data in an Ar and He bath gas separately. A temperature dependent ⟨Δ*E*⟩_d_ was assumed with the following expression

E3 $${\left\langle \Delta E\right\rangle }_{d}=\left({\left\langle \Delta E\right\rangle }_{d,298K}\right){\left(\frac{T\left(K\right)}{298K}\right)}^{n}$$

The values of (⟨Δ*E*⟩_d, 298*K*_) in both Ar and He were fit to experiment. The *n* parameter proved to be too ill-defined by the current fits and was fixed at values of 0.5 in each case.

For these calculations, all internal rotational modes were treated as hindered rotors and in the RO_2_ species, the nonmethyl rotations were explicitly coupled as described previously.^12^ The R and RO_2_ species were characterized at the CCSDT-F12/aug-cc-pVTZ//M062X/6–31 + G** level of theory and all separable and coupled hindered rotor potentials were performed in increments of 30° with relaxed optimizations at the M062X/6–31 + G** level of theory.

It was also important to consider further reaction of the RO_2_ species in the form of internal hydrogen transfer followed by dissociation to form OH, i.e. RO_2_ → QOOH → OH + product. This process will be discussed further in a future publication but for now we have characterized the two internal hydrogen transfer transition states at the CCSDT-F12/aug-cc-pVTZ//M062X/6–31 + G** level of theory and for all fits we have allowed these transition state energy to float. We have assumed both reactions to proceed irreversible to OH formation once internal hydrogen transfer occurs in line with previous calculations.^25^

## 4 Results and Discussion

### 4.1 Kinetics of the Reaction of Cl with MF (R3)

When (COCl)_2_ was photolyzed at 266 nm in the presence of MF and O_2_, OH formation was observed; examples of OH profiles are shown in Figures 1, 3 and the Supporting Information. The OH was formed by the Cl + MF reaction (R3) followed by the R + O_2_ reactions^12,27^ and then OH was removed by diffusion out of the observation volume and reaction with MF (Scheme 1 in Introduction).

**Figure 1:**
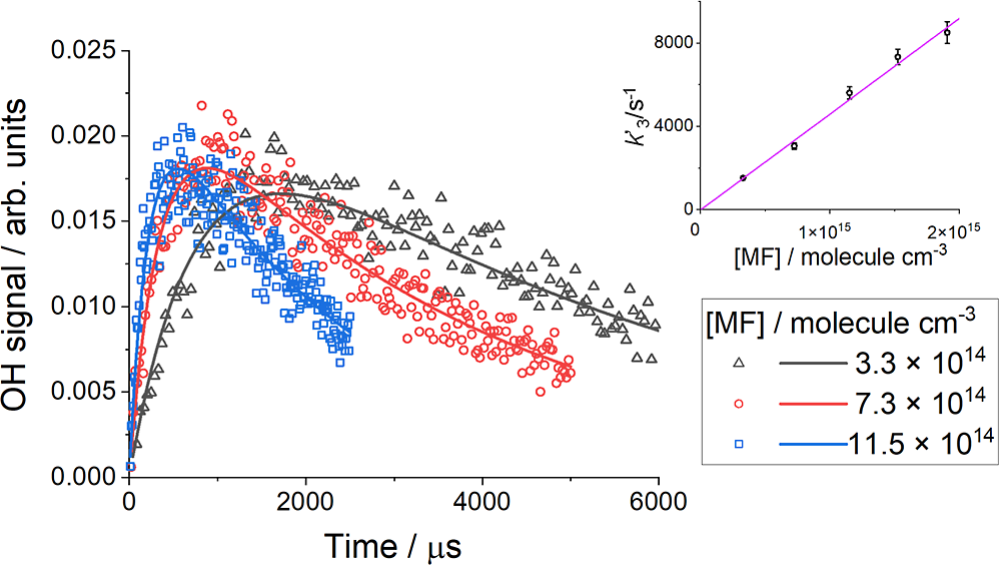
Examples of the OH signal–time profiles used in the simultaneous fit of equation ES1 (Supporting Information) to the experimental data collected for Cl + MF (R3) at 411 K and 19 Torr of O_2_. The inset shows the bimolecular rate coefficient plot obtained by the experiment; *k*_3,Cl+MF_ = (4.4 ± 0.8) × 10^–12^ cm^3^ molecule^–1^ s^–1^ where the error is statistical at 2σ level.

The kinetics of reaction R3 (Cl + MF) were studied from 213 to 420 K. Sufficient oxygen was added such that the R + O_2_ reactions were not rate-determining ([O_2_] = 2 × 10^16^–2 × 10^17^ molecule cm^–3^). Under these conditions the OH signal vs time profiles were biexponential in nature with reaction R3 being the rate-determining step in the formation of OH. Figure 1 shows examples of OH temporal profiles obtained using Cl + MF/O_2_. The experiments were carried out under pseudo-first order conditions and the observed OH kinetics can be described by the biexponential equation ES1 (Supporting Information, S1).

Experimental data collected at each temperature were fitted simultaneously by equation ES1 using the pseudo-first order coefficient for the Cl diffusion out of the measurement volume, *k*′_d(Cl)_ as a common parameter to extract the pseudo-first order rate coefficients, *k*′_3,Cl+MF_ = *k*_3,Cl+MF_[MF], and *k*′_OH loss_. Figure 1 shows examples of results obtained for a range of concentrations of MF at 411 K and 19 Torr. A virtually zero value was obtained for parameter *k*′_d(Cl)_ at all temperatures. The bimolecular rate coefficient *k*_3,Cl+MF_ was derived from the slopes of the plots of *k*′_3,Cl+MF_ vs [MF]. An example bimolecular plot is shown in the inset of Figure 1.

Figure 2 shows the positive temperature dependences for both experimental and calculated *k*_3,Cl+MF_ in the range of the measurements, 213–420 K. The room temperature (294 K) value of *k*_3,Cl+MF_ = (1.8 ± 0.4) × 10^–12^ cm^3^ molecule^–1^ s^–1^, where the error is a combination in quadrature of the statistical error at the 2σ level and 5% systematic error, lies in the range of experimental values reported at room temperature, (1.0–1.8) × 10^–12^ cm^3^ molecule^–1^ s^–1^.^28,30–33^

**Figure 2:**
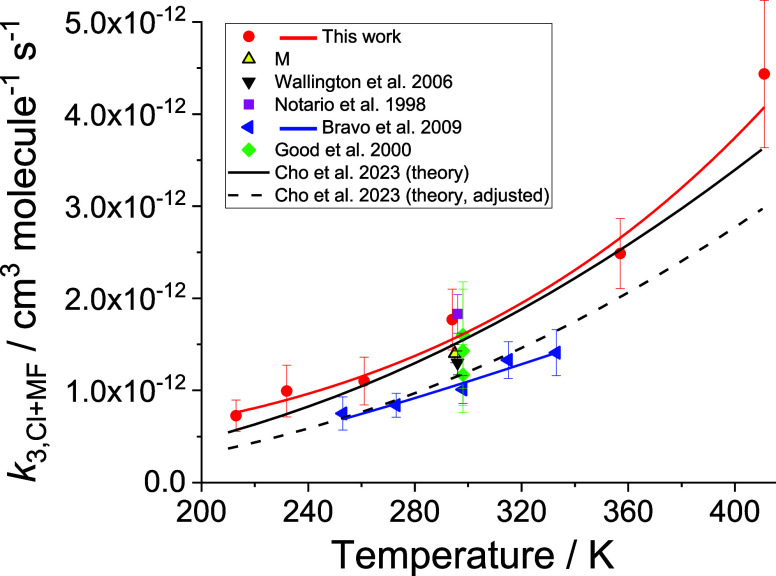
Temperature dependence of *k*_3,Cl+MF_ including literature data. The experimental results of this work (individual points show with 2σ errors) can be characterized as: *k*_3,Cl + MF_ = (2.5 ± 5.9) × 10^–13^ (*T*/298)^4.5^ ± ^2.4^ exp((560 ± 700)/*T*) cm^3^ molecule^–1^ s^–1^. The experimental data obtained by Bravo et al.^33^ (blue triangle) can be described by: *k*_3,Cl + MF_ = (1.7 ± 1.4) × 10^–11^ exp(−(810 ± 250)/*T*). The prediction by the theory study of Cho et al.^19^ before (black solid line) and after the adjustment (black dash line) performed by the authors (see text) are also shown.

The present experimental data can be characterized as: *k*_3,Cl+MF_ = (2.5 ± 5.9) × 10^–13^ (*T*/298)^4.5 ± 2.4^ exp((560 ± 700)/*T*) cm^3^ molecule^–1^ s^–1^ (Figure 2). The other experimental study of the temperature dependence by Bravo et al.^33^ was performed between 253–333 K and measured values for *k*_3,Cl+MF_ lower than the present determinations by 25–40%. The result of these authors’ study at 298 K, (1.0 ± 0.2) × 10^–12^ cm^3^ molecule^–1^ s^–1^, is also lower than all the other experimental values of *k*_3,Cl+MF_ found previously at room temperature^28,30–33^ (Figure 2). The recent calculations of Cho et al.^19^ predicted *k*_3,Cl+MF_ from 200 to 2000 K combining ab initio calculations with master equation analysis. The authors then adjusted the ab initio barrier for reaction R3b to improve the agreement of their predictions with the experimental determinations of the branching ratios in R3 at 296 K.^13,42^ Figure 2 shows both the unadjusted and adjusted *k*_Cl+MF_ vs *T* in the range of the present experiments, 213–411 K. It can be noted that both temperature dependences predicted by Cho et al.^19^ agree with the present result within the expected uncertainty of the theoretical calculations (factor of 1.5) with the unadjusted calculations in a better agreement with this work.

In addition, the temperature dependence of the rate coefficients of the Cl + MF-d1 reaction was measured in the range 210–600 K. Figure S1 in the Supporting Information shows that the ratio of *k*_3,Cl+MF‑d1_ vs *T*: *k*_3,Cl+MF_ vs *T* give a small kinetic isotope effect of *k*_3,Cl+MF_/*k*_3,Cl+MF‑d1_ between 1.2 at 213 K and 1.4 at 411 K.

### 4.2 Kinetics of R + O_2_ Reactions

#### 4.2.1 *k*_1,R+O_2__ Determined from Cl + MF in the Presence of O_2_

These measurements were performed between 213 K and 420 K and at pressures in the range ∼5–100 Torr of N_2_ or Ar. Some additional experiments were performed, generating R from the reaction of OH with MF (Supporting Information, section S2). The concentrations of MF were (0.6–2.5) × 10^16^ molecule cm^–3^ and maintained constant during each experiment while [O_2_] was varied in the typical range of (0.2–1.0) × 10^15^ molecule cm^–3^. Under these conditions, the initial reaction (Cl + MF, R3) was faster than the subsequent R + O_2_ reaction, enabling the determination of the rate coefficient for the R + O_2_ reaction, *k*_1_,_R+O_2__. The chemistry can be described by Scheme 1. The OH kinetic traces were triexponential with an initial slow growth on the time scale of 10–20 μs followed by a more rapid growth on a scale of hundreds of μs and then by a slow decay. Figure 3 shows examples of OH signal–time profiles for MF.

**Figure 3:**
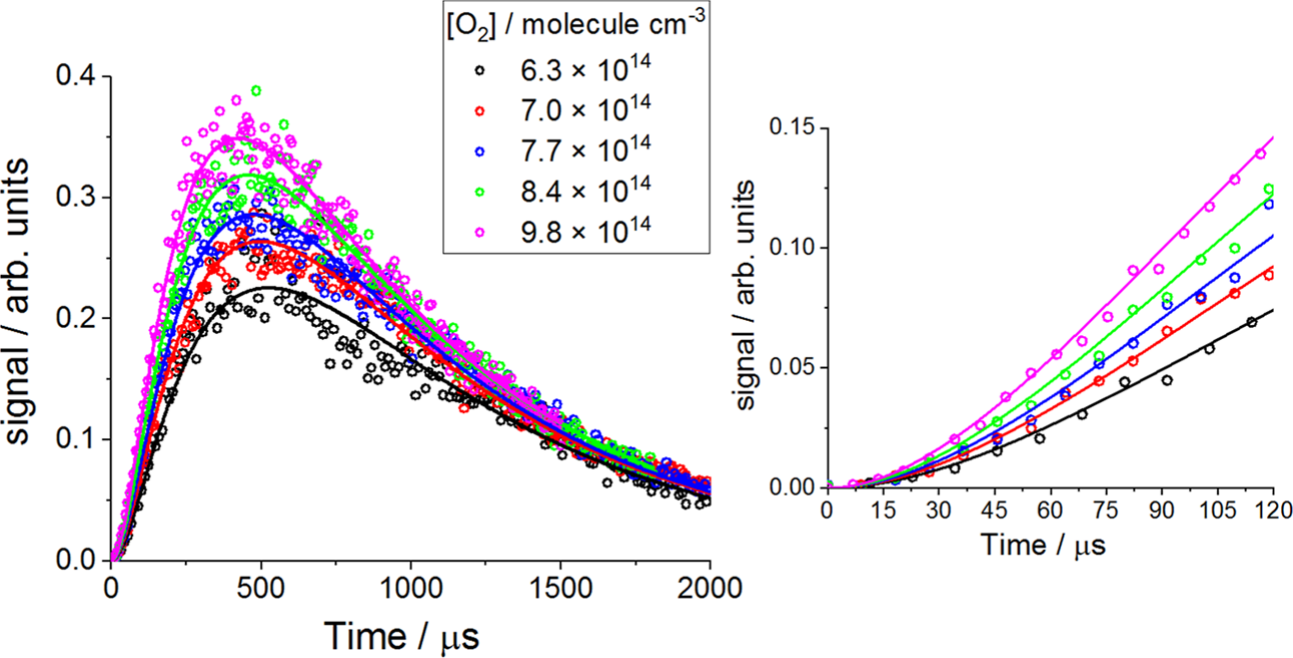
Examples of OH observations (open circles) and results (solid lines) of the simultaneously fit of eq E1 to the data at 294 K and 10 Torr of N_2_. For all traces [MF] = 5.6 × 10^15^ molecule cm^–3^. The fit extracted *k*_1,R+O_2__ = (4.1 ± 0.3) × 10^–12^ cm^3^ molecule^–1^ s^–1^ where the error is statistical at the 2σ level. Data over the first 120 μs have been expanded to illustrate the induction period.

The OH kinetic traces measured at each temperature and pressure were simultaneously fitted to by eq E1 where the terms were given by

$$A=\frac{k_{1,R+O_{2}}^{\prime }k_{3,Cl+MF}^{\prime }}{\left(k_{1,R+O_{2}}^{\prime }-k_{a,Cl+MF}^{\prime }\right)\left(k_{1,R+O_{2}}^{\prime }-k_{OHloss}^{\prime }\right)\left(k_{OHloss}^{\prime }-k_{a,Cl+MF}^{\prime }\right)},,B_{1}=k_{1,R+O_{2}}^{\prime }-k_{OHloss}^{\prime },,B_{2}=k_{OHloss}^{\prime }-k_{3,Cl+MF}^{\prime },,B_{3}=-\left(k_{1,R+O_{2}}^{\prime }-k_{3,Cl+MF}^{\prime }\right),,C_{1}=k_{3,Cl+MF}^{\prime },,C_{2}=k_{1,R+O_{2}}^{\prime }\;\text{and},C_{3}=k_{OHloss}^{\prime }.$$

During the fit, the bimolecular rate coefficient *k*_1,R+O_2__ was treated as a global parameter and [MF] and [O_2_] were constrained to the experimental values. The bimolecular rate coefficient for the Cl + MF reaction (*k*_3,Cl + MF_) was constrained to the value determined in separate experiments (above) and *k*_1,R+O_2__ and *k*′_OH loss_ were floated. Typical fit results are shown in Figure 3 for Cl + MF/O_2_.

As OH was produced by the reactions of O_2_ with both types of radicals formed by R3 (CH_3_OCO and CH_2_OCHO), the kinetic measurements do not allow the separate retrieval of *k*_1a,CH_3_OCO+O_2__ and *k*_1b,CH_2_OCHO+O_2__. At *T* ≤ 294 K the decomposition reactions of CH_3_OCO and CH_2_OCHO were negligible. Thus, *k*_1,R+O_2__ represents a weighted average of the rate coefficients for the CH_2_OCHO + O_2_ and CH_3_OCO + O_2_ reactions. The weights are given by the branching ratios in the initial Cl reaction: *k*_1,R+O_2__ = [*r*_CHO_ × *k*_1a,CH_3_OCO+O_2__ + *r*_CH_3__ × *k*_1b,CH_2_OC(O)H+O_2__], where *r*_CHO_ = $\frac{k_{3a}}{k_{3}}$ and *r*_CH3_ = $\frac{k_{3b}}{k_{3}}$ are the branching ratios for the H-abstraction at the CHO and CH_3_ sites. However, at temperatures above ∼400 K the formate-group centered radical, CH_3_OCO, produced by R3a, decomposes rapidly (R2), while the methyl-centered radical, CH_2_OCHO, formed through R3b, is significantly more stable.^19^ At the highest temperature of ∼420 K used in the experiments with MF (Figure 4a,b), the CH_3_OCO removal was dominated by unimolecular decomposition (*k*_2,dec_ ∼ 10^4^ s^–1^; Supporting Information, section S3). Therefore, at ∼420 K the vast majority of CH_3_OCO radicals decomposed and *k*_1,R+O_2__ becomes ∼*k*_1b,CH_2_OCHO+O_2__.

**Figure 4:**
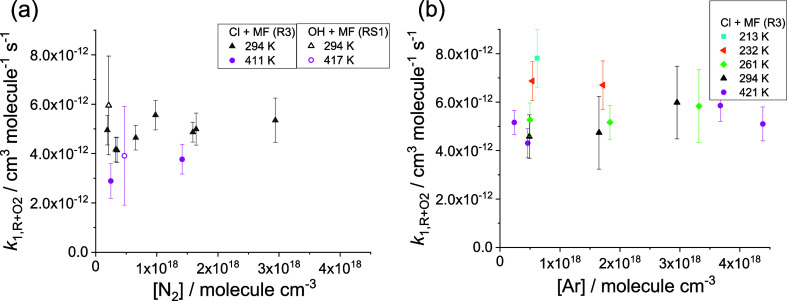
Plots of the bimolecular rate coefficient *k*_1,R+O_2__ vs concentration of bath gas, (a) N_2_ and (b) Ar, at temperatures in the range ∼210–420 K where the radicals were generated by the reactions: Cl + MF (R3) for N_2_ and Ar or OH + MF, N_2_ only. Uncertainties are combinations in quadrature of statistical errors at 2σ level and 5% systematic errors.

Figure 4 shows a summary of *k*_1,R+O_2__ obtained for MF that also includes results generated by using the OH + MF reaction in the presence of O_2_ to generate R (Supporting Information, section S2). The value obtained employing Cl + MF (R3) to generate R at 294 K and 6 Torr of N_2_, *k*_1,R+O_2__ = (5.0 ± 0.4) × 10^–12^ cm^3^ molecule^–1^ s^–1^ has overlapping error bars with the result using OH + MF (RS6) to generate R at identical conditions, *k*_1,R+O_2__ = (6.0 ± 1.9) × 10^–12^ cm^3^ molecule^–1^ s^–1^. The similarity in the values obtained using Cl + MF and OH + MF for R generation, suggests similar branching ratios in the OH and Cl reactions with MF at 294 K, in agreement with previous studies of the branching ratios in the two reactions. The product study of Tyndall et al.,^43^ which used FTIR spectroscopy, reported almost the same branching ratios in the Cl + MF and OH + MF reactions at 296 K, with the abstraction from CH_3_ site roughly as important as the abstraction from CHO site. The result of Tyndall et al. for the Cl + MF reaction agrees with the FTIR measurements performed by Wallington et al.^13^ and Hansen et al.^42^ At 300 K Robertson et al.^12^ determined that the branching ratios in reaction RS6 (OH + MF) are approximately 50:50 combining experiments with calculations, in agreement with the result of earlier work^13,42,43^ and the prediction of Wu et al.,^44^ *r*_CHO(RS6)_ = 0.6 and *r*_CH_3_(RS6)_ = 0.4.

The results for *k*_1,R+O_2__ show little pressure dependence at *T* ≤ 294 K but suggest a potential, weak pressure dependence at *T* > 294 K (Figure 4). Our MESMER simulations suggest that even at 213 K, neither reaction is quite at its high-pressure limit at ∼10 Torr, Ar (∼98% for CH_2_OCHO and 92% for CH_3_OCO); however, the scatter of the experimental data are such that such subtle pressure dependence will be difficult to observe. The kinetics of the R + O_2_ reactions exhibit a negative temperature dependence, as would be expected for association reactions.

#### 4.2.2 *k*_1a,CH_3_OCO+O_2__ and *k*_1b,CH_2_OCDO+O_2__ Determined from Cl + MF-d1 in the Presence of O_2_

To determine *k*_1a,CH_3_OCO+O_2__ and *k*_1b,CH_2_OCDO+O_2__, experiments were performed using the Cl reactions with partially deuterated MF (CH_3_OCDO, MF-d1) in the presence of O_2_. The parameter *k*_1a,CH_3_OCO+O_2__ was obtained using the measurements of OH produced through the reaction of the formate centered radicals with O_2_ with elimination of OH from CH_3_OC­(O)­O_2_*, while *k*_1b,CH_2_OCDO+O_2__ was obtained by following the OD formed via the reaction of the alkyl-group centered radicals with O_2_ (Scheme 2) and subsequent OD elimination from O_2_CH_2_OCDO*. The formate C–H and C–D bonds in CH_2_OCHO and CH_2_OCDO will be spectators in the association with O_2_ to form OOCH_2_OC­(H/D)O and we therefore assume a negligible kinetic isotope effect, i.e. *k*_1b,CH_2_OCHO+O_2__ ≅ *k*_1b,CH_2_OCDO+O_2__.

**Scheme 2:**
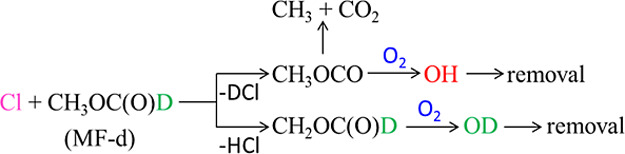


The Cl + MF-d1 experiments were performed in the range 213–472 K in Ar or He. In all experiments the concentration of added oxygen was typically varied in the range (0.2–2.0) × 10^15^ molecule cm^–3^ making the R + O_2_ reaction the rate determining step. As the unimolecular decomposition of CH_2_OCDO radicals was minor under all the experimental conditions, the OD kinetic profiles were analyzed using eq E1 with the terms given above, describing the analysis of the kinetic data generated for Cl + MF.

At *T* ≤ 294 K the unimolecular decomposition of the CH_3_OCO radicals was negligible (Supporting Information, section S3) and, hence the OH temporal profiles obtained using the Cl + MF-d1/O_2_ reactions were analyzed using eq E1. However, in the experiments at *T* > 294 K for MF-d1 the decomposition reaction of CH_3_OCO impacted the measurements. To fit the OH kinetic traces at higher temperatures, the following expressions for the terms of the eq E1 were derived, which incorporate the rate coefficient for the decomposition reaction of CH_3_OCO, *k*_2,dec_

$$A=\left[k_{1a,{CH}_{3}OCO+O_{2}}^{\prime }k_{3,Cl+MF-d1}^{\prime }\right]/\left[\left(k_{1a,{CH}_{3}OCO+O_{2}}^{\prime }+k_{2,dec}-k_{3,Cl+MF-d1}^{\prime }\right)\left(k_{1a,{CH}_{3}OCO+O_{2}}^{\prime }+k_{2,dec}-k_{OHloss}^{\prime }\right)\left(k_{OHloss}^{\prime }-k_{3,Cl+MF-d1}^{\prime }\right)\right]\left[{Cl}_{0}\right],$$

$$B_{1}=k_{1a,{CH}_{3}OCO+O_{2}}^{\prime }+k_{2,dec}-k_{OHloss}^{\prime },,B_{2}=k_{OHloss}^{\prime }-k_{3,Cl+MF-d1}^{\prime },,B_{3}=-\left(k_{1a,{CH}_{3}OCO+O_{2}}^{\prime }+k_{2,dec}-k_{3,Cl+MF-d1}^{\prime }\right),,C_{1}=k_{3,Cl+MF-d1}^{\prime },,C_{2}=k_{1a,{CH}_{3}OCO+O_{2}}^{\prime }+k_{2,dec},\text{and},C_{3}=k_{OHloss}^{\prime },$$

here [Cl]_0_ is the initial concentration of Cl that is converted into OH, *k*′_1a,CH_3_OCO+O_2__ = *k*_1a,CH_3_OCO+O_2__.[O_2_], *k*′_3,Cl+MF‑d1_ = *k*_3,Cl+MF‑d1_.[MF-d1] and *k*′_OH loss_ is the pseudo-first order rate coefficient for the OH loss by reaction with MF-d1 and diffusion out of the probe volume.

Figure 5 shows examples of OH and OD kinetic measurements and results obtained at 397 K by fitting eq E1 to the observations. Each of the sets of data, OH and OD, collected at each temperature and pressure, were fitted simultaneously. During the fit, [MF-d1] and [O_2_] were constrained to the measured values and the rate coefficients *k*_1a,CH_3_OCO+O_2__ and *k*_2,dec_ (OH data) and *k*_1b,CH_2_OCDO+O_2__ (OD data) were extracted as common parameters. The rate coefficient *k*_3,Cl+MF‑d1_ was treated as a global parameter and constrained to the values determined in separate experiments that employed higher [O_2_] = (2–4) × 10^16^ molecule cm^–3^ (Section S1).

**Figure 5:**
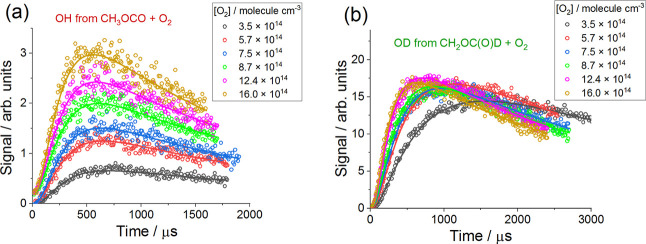
Examples of observations (open circles) and results (solid lines) of the simultaneous fit of eq E1 to the (a) OH data (resulting from abstraction at formate, CDO, site) and (b) OD data (resulting from abstraction at the methyl site) at 397 K and 10 Torr of He. For all traces [MF-d1] = 3.1 × 10^15^ molecule cm^–3^ and [(COCl)_2_] = 1.5 × 10^14^ molecule cm^–3^ and the laser fluence was 50 mJ cm^–2^ pulse^–1^. The fit to the OH measurements (a) extracted *k*_1a,CH_3_OCO+O_2__ = (7.0 ± 1.0) × 10^–13^ cm^3^ molecule^–1^ s^–1^ and *k*_2,dec_ = (5500 ± 280) s^–1^. The fit to the OD data (b) gives *k*_1b,CH_2_OCDO+O_2__ = (4.5 ± 0.2) × 10^–12^ cm^3^ molecule^–1^ s^–1^. Errors are statistical at 2σ level.

Under all conditions in the experiments using lower oxygen concentrations, increasing [O_2_], while [MF-d1] and [(COCl)_2_] were maintained constant, resulted in an increase in the fluorescence signal as the rate of the R + O_2_ reaction became greater (Figure 5). At *T* ≤ 294 K the percentage increases in the maxima of the OH and OD signals with [O_2_] were similar. However, at higher temperatures the decomposition of the CH_3_OCO radicals (R2) had an effect on the concentration–time profiles of OH as the CH_3_OCO + O_2_ reaction was in competition with CH_3_OCO decomposition. Therefore, increasing the O_2_ present in the system led to more interception of CH_3_OCO by O_2_. In contrast, the dissociation reaction of CH_2_OCDO radicals was negligible at all experimental temperatures and thus did not impact the OD kinetic profiles. Therefore, at higher temperatures the percentage increase in the maximum of the OH signal with [O_2_] was more significant than the percentage growth of the maximum of the OD signal with [O_2_].

Table 1 and 2 show *k*_1a,CH_3_OCO+O_2__ and *k*_1b,CH_2_OCDO+O_2__ determined as a function of temperature in Ar and He, respectively. A negative temperature dependence was found for both isomer specific rate coefficients. The isomer specific rate coefficients at 294 K and 15 Torr of Ar: *k*_1a,CH_3_OCO+O_2__ = (3.0 ± 0.8) × 10^–12^ cm^3^ molecule^–1^ s^–1^, *k*_1b,CH_2_OCDO+O_2__ = (6.1 ± 0.6) × 10^–12^ cm^3^ molecule^–1^ s^–1^ (Table 1) can be compared with the total rate coefficient *k*_1,R+O_2__. As *k*_R+O_2__ = *r*_C(O)H_× *k*_1a,CH_3_OCO+O_2__ + *r*_CH_3__ × *k*_1b,CH_2_OCHO+O_2__ and *r*_C(O)H_ = *r*_CH_3__ = 0.5,^13,44^ then *k*_1,R+O_2__ = (4.6 ± 0.6) × 10^–12^ cm^3^ molecule^–1^ s^–1^. This calculated value of *k*_1,R+O_2__ from the isotopomer specific measurements is in excellent agreement with *k*_1,R+O_2__ = (4.6 ± 0.9) × 10^–12^ cm^3^ molecule^–1^ s^–1^ measured in the Cl + MF/O_2_ experiments at identical conditions (Figure 4b) presented in the previous subsection. Under all conditions *k*_1a,CH_3_OCO+O_2__ was lower than *k*_1b,CH_2_OCDO+O_2__. Similarly, it has been found that the CH_3_CO + O_2_ reaction is slower than the CH_2_OCH_3_ + O_2_ reaction under a range of conditions,^45,46^ i.e. the addition of O_2_ to the methyl-centered radical where the CH_2_ group is single bonded to an O atom is faster than the reaction of the carbonyl-centered radical with O_2_.

**Table 1:** Bimolecular Rate Coefficients *k*_1a,CH_3_OCO+O_2__ and *k*_1b,CH_2_OCDO+O_2__ Measured Using Ar Bath Gas

*T*/K	[Ar] × 10^–18^/molecule cm^–3^	*k*_1a,CH_3_OCO+O_2__ × 10^a^/cm^3^ molecule^–1^ s^–1^	*k*_1b,CH_2_OCDO+O_2__ × 10^12^ ^a^/cm^3^ molecule^–1^ s^–1^
213	0.4	5.9 ± 0.8	8.3 ± 2.7
233	0.5	5.4 ± 1.5	8.8 ± 1.6
260	0.5	4.2 ± 1.4	7.1 ± 2.5
294	0.5	3.0 ± 0.8	6.2 ± 0.8
294	0.5		6.0 ± 0.4
294	2.7		7.7 ± 1.2
348	0.3	1.8 ± 0.4	
373	0.3		4.6 ± 0.4
398	0.2	1.4 ± 0.4	3.2 ± 0.3
398	0.2		3.4 ± 0.7
398	0.3	1.7 ± 0.6	4.0 ± 0.3
418	0.4		2.6 ± 0.6
418	2.0		3.4 ± 0.7
423	0.2	1.6 ± 1.0	4.0 ± 0.5
423	0.3		2.9 ± 0.5
472	0.2	0.9 ± 0.8	2.9 ± 0.3

a Uncertainties are combinations in quadrature of statistical errors at 2σ level and estimated 5% systematic errors.

**Table 2:** Bimolecular Rate Coefficients *k*_CH_3_OCO+O_2__ and *k*_CH_2_OCDO+O_2__ Measured Using He Bath Gas

*T*/K	[He] × 10^–18^/molecule cm^–3^	*k*_1a,CH_3_OCO+O_2__ × 10^12^ ^a^/cm^3^ molecule^–1^ s^–1^	*k*_1b,CH_2_OCDO+O_2__ × 10^12^ ^a^/cm^3^ molecule^–1^ s^–1^
398	0.2	0.7 ± 0.2	3.8 ± 0.7
398	1.2	1.6 ± 0.4	5.6 ± 0.6
430	0.2	0.6 ± 0.2	2.9 ± 0.3
430	1.1	1.3 ± 0.4	4.9 ± 0.4
464	0.2		3.2 ± 0.3

a Uncertainties are combinations in quadrature of statistical errors at 2σ level and 5% systematic errors.

To investigate the pressure dependence of *k*_1a,CH_3_OCO+O_2__ and *k*_1b,CH_2_OCDO+O_2__ at higher temperatures, Cl + MF-d1/O_2_ experiments were performed in He, in the range ∼(400–460) K. Tables 1 and 2 show that at [M] ∼2 × 10^17^ molecule cm^–3^, *k*_1b,CH_2_OCDO+O_2__(He) is in the range of values obtained for *k*_1b,CH_2_OCDO+O_2__(Ar) while *k*_1a,CH_3_OCO+O_2__(He) < *k*_1a,CH_3_OCO+O_2__(Ar). On increasing pressure from 11 to 50 Torr of helium, *k*_1b,CH_2_OCDO+O_2__(He) increases by a factor of 1.5 ± 0.2 (398 K) and 1.7 ± 0.1 (430 K) and *k*_1a,CH_3_OCO+O_2__(He) increases by a factor of 2.3 ± 0.4 at both 398 and 430 K. The results suggest that at these higher temperatures, *k*_1a,CH_3_OCO+O_2__ is further into the falloff region than *k*_1b,CH_2_OCDO+O_2__.

#### 4.2.3 Modeling the Pressure and Temperature Dependence of R1a and R1b

Figure 6 shows the stationary point energies for the RO_2_ adducts and the internal H transfer (RO_2_ → QOOH) saddle points for each R + O_2_ reaction.

**Figure 6:**
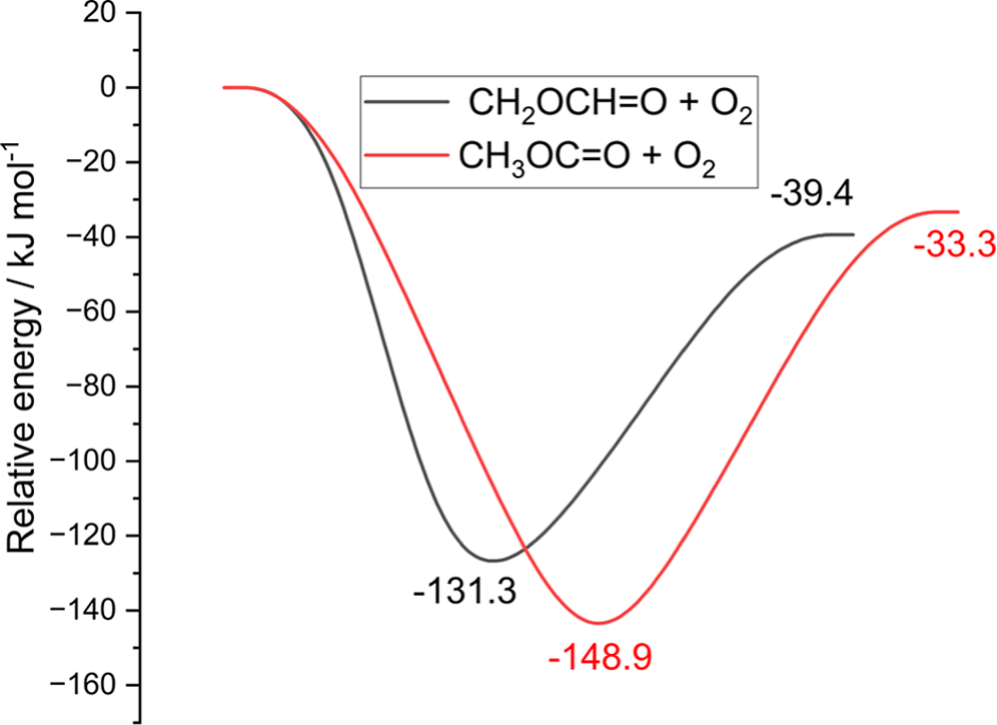
Potential energy surface showing the relative energies of the RO_2_ species and the internal hydrogen transfer transition states to form QOOH used in the current calculations. For this work, reaction was considered complete once hydrogen migration had occurred. The energies were calculated at the CCSDT-F12/aug-cc-pVTZ//M062X/6–31 + G** level of theory.

The MESMER model readily fits the experimental data with both Ar (Figure 7) and He (Figure S6 and Section S4) bath gases. Given the relatively small amount of data and the number of adjustable parameters, many of the fitted parameters are ill-defined by the Levenberg–Marquardt fits with large associated errors. The fitted parameters are given in Table 3 for both R + O_2_ reactions. These fitting results suggest that neither R + O_2_ reaction has quite reached its asymptotic high pressure limit and Figure S7 plots the predicted rate coefficients from the optimized MESMER models for both R + O_2_ reactions as a function of total [Ar] at 213 K. The MESMER modeling predicts that neither reaction has reached the high pressure at the experimental conditions although the CH_2_OCHO + O_2_ case is closer to its high-pressure limit. This modeling does predict a larger high pressure limiting rate coefficient in the CH_2_OCHO + O_2_ case, as supported by our qualitative CASPT2 calculations, see below, however the fitted ILT *A* factors for the two reactions do overlap within combined errors. A more detailed investigation of the wider kinetics of these systems will be undertaken in the future publication.

**Figure 7:**
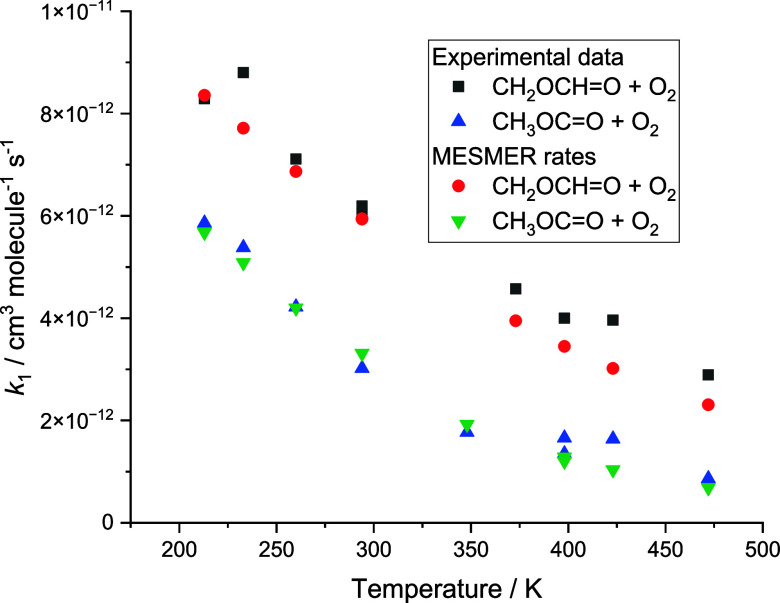
Comparison of experimental and MESMER rate coefficients for the CH_3_OCO + O_2_ and the CH_2_OCHO + O_2_ reactions in Ar.

**Table 3:** Parameters Returned from MESMER Fitting to the Experimental Data^a^

parameter	CH_2_OCHO + O_2_	CH_3_OCO + O_2_
TS1/TS2/kJ mol^–1^	–38 ± 12	–26.2 ± 2.1
⟨Δ*E*⟩_d_,298Ar	800 ± 2800	600 ± 380
⟨Δ*E*⟩_d_,298He	280 ± 700	127 ± 57
ILT *A*/10^–12^molecule^–1^ cm^3^ s^–1^	6.6 ± 1.1	4.4 ± 1.3
ILT *n*	–0.82 ± 0.56	–1.14 ± 0.83

a – errors are 2σ.

A semiqualitative rationale for the enhanced reactivity of CH_2_OCDO over CH_3_OCO with O_2_ comes from our calculations. Despite not being fully quantitative, which would require both corrections corresponding to higher levels of theory and phase space integrations to accurately describe the transitional modes, the calculations show that for the CH_3_OCO + O_2_ case, the potential is less attractive at longer reagent separations.

Additionally, as shown in Figure 8, all orientations of CH_3_OCO + O_2_, apart from one, exhibit barriers to association that is not replicated in the CH_2_OCHO + O_2_ case, indicating an extra conformational ‘tightness’ in the CH_3_OCO + O_2_ case and the lower *A* factor.

**Figure 8:**
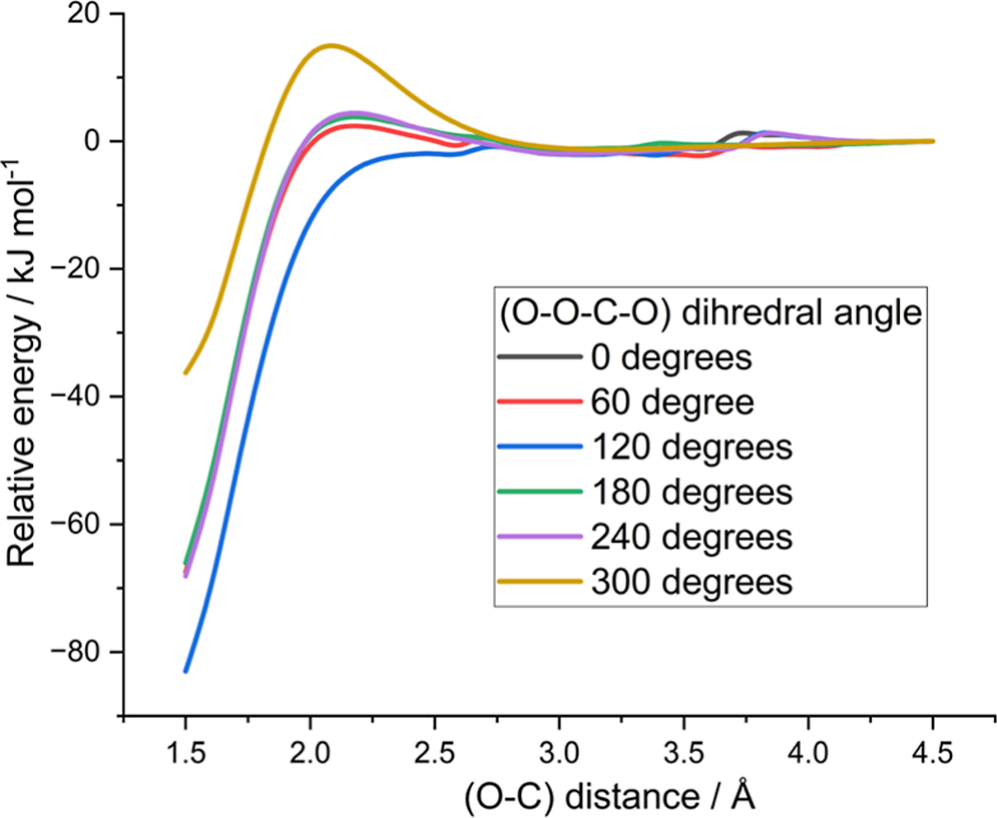
Association curves showing the potential energy as a function of O–C (where C is the radical center on the R) for the CH_3_OCO + O_2_ at different fixed values of the OO-CO torsion angle determining the relative orientation of the two approaching moieties. These energies are taken from relaxed scans at the rs2/aug-cc-pVDZ level of theory.

### 4.3 CH_3_OCO Decomposition

Analysis of the OH data at higher temperatures also returned the rate coefficient for the unimolecular decomposition of CH_3_OCO (Scheme 2, R2) in the range of 348–472 K. A positive temperature dependence was observed for *k*_2,dec_. The potential energy surface computed for the decomposition process at the CCSD­(T)-F12/aug-cc-pVDZ//M062X/6–31 + G** level of theory give a barrier of 60.3 kJ mol^–1^ for CH_3_OCO decomposition with an estimated uncertainty of 4 kJ mol^–1^. The result is in good agreement with previous work (Table S1) and more details on the calculations can be found in Section 3 of the Supporting Information. Our calculations are less sophisticated than those of Cho et al.^19^ and therefore when fitting to *k*_2,dec_ using MESMER,^29^ the decomposition barrier was fixed at the ANL0F″ barrier height of 57.3 kJ mol^–1^ calculated previously.^19^ Energy transfer values <Δ*E*>_d_ of <Δ*E*>_d_(Ar) = (110 ± 30) × (*T*/298)^(0.0 ± 0.7)^ cm^–1^ and <Δ*E*>_d_(He) = (34.4 ± 6.2) × (*T*/298)^(1.0 ± 0.5)^ cm^–1^ provided good fits to the experimental data (Figure S6). Further details on the fitting are given in the online Supporting Information.

### 4.4 Implications

Results for the rate coefficients *k*_1,R+O_2__ and *k*_2,dec_ are important for modeling studies of atmospheric chemistry and combustion. The R + O_2_ reaction promotes autoxidation of the fuel radicals, whereas radical decomposition leads to smaller radicals with different routes to chain branching. Figure 9 compares the pseudo-first-order loss of CH_3_OCO by reaction with O_2_, *k*′_1a,CH_3_OCO+O_2__ = *k*_1a,CH_3_OCO+O_2__ × [O_2_], with the loss of CH_3_OCO by unimolecular decomposition (R2), *k*_2,dec_, at 1 bar of air in the range 300–900 K. The losses were determined by using MESMER calculations with the average downward energy transferred <Δ*E*>_d_(air) = *m* × <Δ*E*>_d_(Ar), where *m* = 1.0, 1.5 and 2.0 respectively and <Δ*E*>_d_(Ar) given by the MESMER fit to the experimental data (Supporting Information, section S3): 363 ± 140 cm^–1^ for the CH_3_OCO + O_2_ reaction and 110 ± 30 cm^–1^ for R2. As the CH_3_OCO + O_2_ reaction is close to the high pressure limit at 1 bar of air, *k*’_1a,CH3OCO+O2_ shows little sensitivity to the parameter *m* and the variation with *m* is on the order of the experimental uncertainty. The rate coefficient *k*_2,dec_ is in the falloff region at 1 bar of air and, thus is sensitive to the change in *m*. With increasing temperature, *k*_2,dec_ becomes closer to the low pressure end of the falloff region and, thus increasingly sensitive to the value of <Δ*E*>_d_(air) considered in the analysis. The percentage deviation of *k*_2,dec_(*m* = 2.0) from *k*_2,dec_(*m* = 1.0) is 30% at 300 K and 200% at 900 K. Therefore, the uncertainty in <Δ*E*>_d_(air) is the main source of error in *k*_2,dec_.

**Figure 9:**
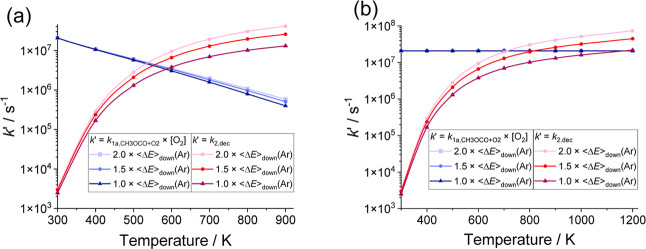
Comparison of the pseudo-first-order loss of CH_3_OCO by reaction with O_2_ (R1a; blue lines and points) with the loss of CH_3_OCO by unimolecular decomposition (R2; red lines and points) at 1 bar of air as a function of temperature. (a) The rate coefficients for both reactions were calculated by MESMER analysis using the literature barrier for R2, 57.3 kJ mol^–1^.^19^ <Δ*E*>_d_(air) = 2.0 × <Δ*E*>_down_(Ar), 1.5 × <Δ*E*>_d_(Ar) and 1.0 × <Δ*E*>_d_(Ar), where <Δ*E*>_d_(Ar) = 363 ± 140 cm^–1^ for CH_3_OCO + O_2_ and <Δ*E*>_d_(Ar) = 110 ± 30 cm^–1^ for reaction R2. (b) R2 treated as above, but fixed value of *k*_1,R+O_2__, based on our measured *k*_1,R+O_2__ 298 K.

Figure 9 shows that the reaction with O_2_ dominates CH_3_OCO removal under atmospheric conditions, while CH_3_OCO decomposition becomes more significant at low-temperature combustion temperatures. Between ∼550 and 650 K *k*_2,dec_ = *k*′_1a,CH_3_OCO+O_2__ (1 bar air). Figure 9 also shows that, for the current system, constrained with experimental data, parameters associated with the radical dissociation channel are key in the determining the equivalence temperature for decomposition (chain propagation and formation of smaller radicals) vs reaction with O_2_ (potential for chain branching). However, this study also shows that there can be significant variations in R + O_2_ rate coefficients depending on the structure of the radical R. Additionally, some models assume that the R + O_2_ reaction is both temperature and pressure independent, e.g^.^ ^47^ and comparison of Figure 9a,b, shows that such assumptions can have significant implications in determining equivalence temperatures for decomposition and reaction with O_2_.

The competition between dissociation and reaction with O_2_ is particularly important when R = QOOH, one of the key species in LTC chain branching. Although some studies on QOOH species have been carried out, e.g.^48,49^ QOOH species are difficult to generate in a controlled manner. Therefore, studies on a wider range of radical + O_2_ species, particularly when the radical isomers are selectively isolated, will provide data to allow for more accurate predictions of QOOH + O_2_ rate coefficients.

## 5 Conclusions

The kinetics of the association reaction of O_2_ with the radicals formed through the Cl + MF reaction were studied measuring the temporal profiles of OH generated by chemical activation using the pulsed laser flash photolysis–laser induced fluorescence technique. This method provided weighted averages of *k*_1a,CH_3_OCO+O_2__ and *k*_1b,CH_2_OCHO+O_2__ with the weights given by the branching ratios in the initial Cl reaction with MF in the range 213–420 K and between 5–100 Torr of N_2_ or Ar.

The measurements of the OH and OD fluorescence signals vs time obtained in experiments using Cl + MF-d1 in the presence of O_2_ enabled the determination of the isomer specific rate coefficients *k*_1a,CH_3_OCO+O_2__ and *k*_1b,CH_2__ _OCDO+O___2__ from 213 to 472 K and typically at pressures around 10 Torr of Ar/He. Negative temperature dependences for the rate coefficients were observed and the rate coefficients showed little pressure dependence. The results of the isomer specific studies were in agreement with the values of *k*_1,R+O_2__ found by the Cl + MF experiments. For all conditions *k*_1b,CH_2_OCDO+O_2__ > *k*_1a,CH_3_OCO+O_2__ and this observation is qualitatively supported by calculations which show a more attractive surface for the CH_2_OCDO + O_2_ reaction and greater entropic constraints on the transition state for the CH_3_OCO + O_2_ reaction.

The analysis of the kinetic traces at higher temperatures also returned the rate coefficient of the dissociation reaction of CH_3_OCO, *k*_2,dec_, in the range 348–470 K. The results showed the expected positive dependence of *k*_2,dec_ on temperature. MESMER analysis, using the energy barrier found by ANL0F″ calculations previously, 57.3 kJ mol^–1^,^19^ provided a good fit to the data with <Δ*E*>_d_(Ar) = (110 ± 30) × (*T*/298)^(0.0 ± 0.7)^ cm^–1^ and <Δ*E*>_d_(He) = (34.4 ± 6.2) × (*T*/298)^(1.0 ± 0.5)^ cm^–1^.

This work shows that under atmospheric conditions the reaction with O_2_ dominates the fate of CH_3_OCO. At 1 bar of air the loss of CH_3_OCO by reaction with O_2_ is in competition with the CH_3_OCO removal by decomposition from 550 to 650 K. The CH_3_OCO dissociation dominates above 650 K. Further isomer specific studies of R + O_2_ reactions would help provide more accurate predictions of QOOH + O_2_ rates, key steps in LTC chain branching.

## Supplementary Material



## Notes

The kinetics studies of the Cl + CH_3_OCDO kinetics (section S1), details on the determination of R + O_2_, generating R via OH kinetics (S2), detail on calculations of CH_3_OCO decomposition (S3) and the pressure and temperature dependence of R3a and R3b (S4) (PDF)
