# Supplementary material for: Kinetics of the O2 Reactions with CH3OCO and CH2OCHO Formed from Methyl Formate
Source: J Phys Chem A. 2026 Apr 16;130(17):3451–61. doi: 10.1021/acs.jpca.6c01296 (PMC13137248; doi:10.1021/acs.jpca.6c01296)
Supplement: Supplementary file 1 [file jp6c01296_si_001.pdf]

Kinetics of the O_2 Reactions with CH_3OCO and CH_2OCHO Formed from Methyl Formate

Lavinia Onel [1,*], Robin Shannon[1], Niamh C. K. Robertson[1], Mark A. Blitz[1,2], Daniel Stone[1], Paul W. Seakins[1,*]

[1] School of Chemistry, University of Leeds, Leeds, LS2 9JT, United Kingdom

[2] National Centre for Atmospheric Science, University of Leeds, Leeds, LS2 9JT, United Kingdom

Contents

S1 - Kinetics of the reaction Cl with MF/MF-d1
S2 - Determination of k_{1R+O2} using OH + MF/O_2
S3 - Kinetics of the unimolecular decomposition of CH_3OCO
S4 - Pressure and Temperature dependence of $k_{CH3OCO+O2}$ over $k_{CH2OCDO+O2}$

S1 Kinetics of the reaction Cl with MF/MF-d1

The experiments were performed under condition where $k'_{3,Cl+MF} << k'_{1,R+O2}$ by using high [O_2], ~$10^{16} – 10^{17}$ molecule cm^{-3}. Under such conditions, the rate coefficient of the Cl reaction with MF/MF-d1 was the rate-determining step in the formation of OH and determined by fitting equation ES1 to the kinetic data.

$$[OH] = \frac{k'_{3,Cl+MF}\,[Cl]_0}{k'_{3,Cl+MF} + k'_{d(Cl)} - k'_{OH\,loss}}\left[\exp(-k'_{OH\,loss}t) - \exp\left[-(k'_{3,Cl+MF} + k'_{d(Cl)})t\right]\right] \quad (ES1)$$

Here $[Cl]_0$ is the initial concentration of Cl that is converted into OH, $k'_{3,Cl+MF}$ is the pseudo-first order rate coefficient for reaction R3 (main text) or the Cl + MF-d1 reaction, $k'_{3,Cl+MF} = k_{3,Cl+MF}$[MF] or $k'_{3,Cl+MF-d1} = k_{3,Cl+MF-d1}$[MF-d1], $k'_{d(Cl)}$ is the pseudo-first order coefficient for the Cl diffusion out of the measurement volume and $k'_{OH\,loss}$ is the pseudo-first order rate coefficient for OH loss.

Fitting modified Arrhenius expressions to the experimental data (Fig. S1) resulted in: $k_{3,Cl+MF} = (2.5 \pm 5.9) \times 10^{-13}\,(T/298)^{4.5\pm2.4}\exp((560 \pm 700)/T)$ cm^3 molecule^{-1} s^{-1} and $k_{3,Cl+MFd1} = (4.2 \pm 9.5) \times 10^{-13}\,(T/298)^{3.6\pm1.9}\exp((350 \pm 660)/T)$ cm^3 molecule^{-1} s^{-1}. The fit results correspond to a kinetic isotope effect of $k_{3,Cl+MF}/k_{3,Cl+MF-d1}$ between 1.2 at 213 K and 1.4 at 411 K.

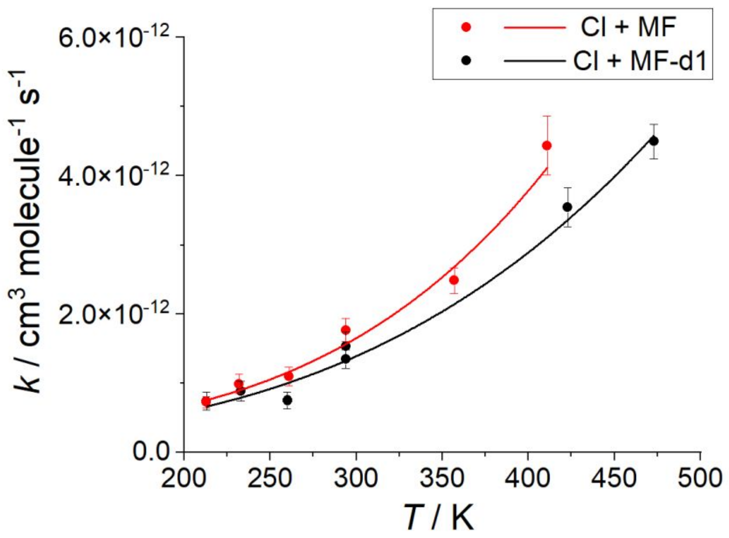

Figure S1. Temperature dependence of $k_{3,Cl+MF}$ and $k_{3,Cl+MF\text{-}d1}$. The error represents the sum in quadrature of the statistical error at the 1σ level and a 5% systematic error. The experimental results of this work can be characterized as: $k_{3,Cl+MF} = (2.5 \pm 5.9) \times 10^{-13} (T/298)^{4.5 \pm 2.4} \exp((560 \pm 700)/T)$ cm^3 molecule^{-1} s^{-1} (red line) and $k_{3,Cl+MF\text{-}d1} = (4.2 \pm 9.5) \times 10^{-13} (T/298)^{3.6 \pm 1.9} \exp((350 \pm 660)/T)$ cm^3 molecule^{-1} s^{-1} (black line).

S2 Determination of $k_{1,R+O2}$ using OH + MF/O_2

The OH kinetics in the OH + MF/O_2 system was also explored to derive $k_{1,R+O2}$. Using relatively low O_2, typically in the range (5–30) × 10^{13} molecule cm^{-3}, the temporal decays of OH were biexponential, with an initial fast decay describing the OH + MF reaction (RS6) followed by a slower decay containing information about the regeneration of OH through the R + O_2 reaction. Figure S2 shows examples of OH temporal decays at 381 K and 6 Torr of N_2. The inset of Fig. S2 shows that increasing [O_2] the tail of the biexponential decay became faster, demonstrating that the R + O_2 reaction was the rate-determining step in the OH regeneration. When [O_2] was above ~5 × 10^{14} molecule cm^{-3}, the two components of the biexponential decay merged in a single exponential, as R + O_2 was no longer rate-determining and, thus $k_{1,R+O2}$ could no longer be derived by the kinetic analysis.

$$OH + CH_3OCHO \rightarrow CH_3OCO + H_2O \quad \text{(RS6a)}$$

$$OH + CH_3OCHO \rightarrow CH_2OCHO + H_2O \quad \text{(RS6b)}$$

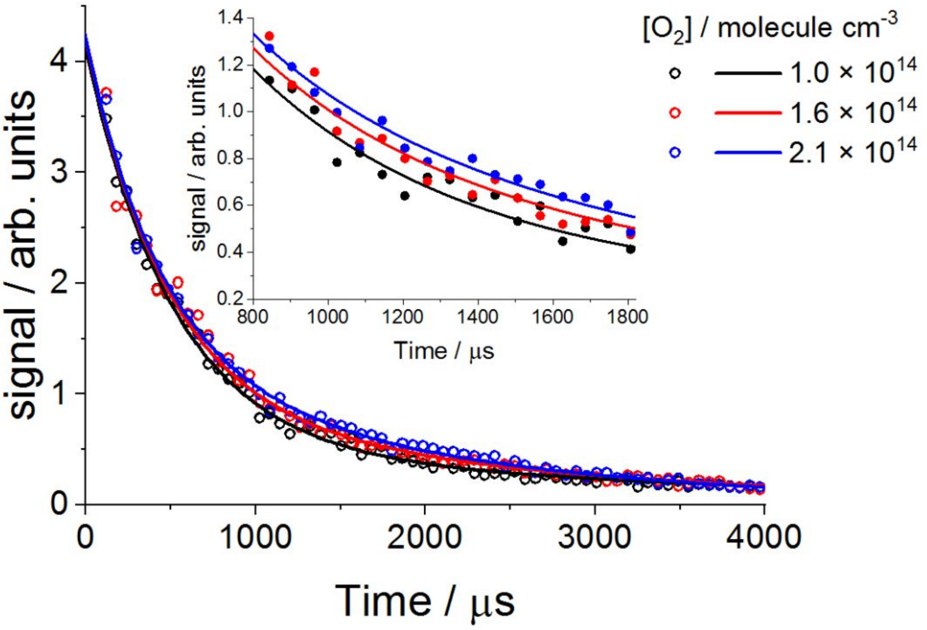

Figure S2. Examples of OH signal temporal decays collected using OH + MF/O_2 at 381 K and 6 Torr of N_2. For all traces [MF] = 4.0 × 10^{15} molecule cm^{-3}. The simultaneous fit of seven kinetic decays returned $k_{1,R+O2}$ = (4.3 ± 0.8) × 10^{-12} cm^3 $molecule^{-1}$ s^{-1} where the error is statistical at the 1σ level. The figure inset shows that the tail of the kinetic decay increased with increasing [O_2].

The OH decays were analysed using the solution of the rate equations for the reactions shown in Scheme S1 (equation ES2).

$$[\mathrm{OH}] = \frac{[\mathrm{OH}]_0}{\lambda_+ - \lambda_-}\left[\left(k'_{\mathrm{RS6}} + k'_{\mathrm{loss}} + \lambda_+\right)\exp\left(\lambda_- t\right) - \left(k'_{\mathrm{RS6}} + k'_{\mathrm{loss}} + \lambda_-\right)\exp\left(\lambda_+ t\right)\right],$$

(ES2)

where $\lambda_{+,-}$=[-M1 ± ($M1^2$ - 4M2)$^{1/2}$]/2, M1 = k_{loss} + k'_{RS6} + k'_{regen} + k'_{stab} and M2 = (k_{loss} + k'_{RS6}) (k'_{regen} + k'_{stab}) - $k'_{RS6}k'_{regen}$. Here k'_{RS6} = k_{RS6} [MF], k'_{regen} = $k_{regen}[O_2]$ and k'_{stab} = $k_{stab}[O_2]$ are the pseudo-first order rate coefficients for reaction RS6, the OH regeneration through R + O_2 and the R species reacting with O_2 to form a collisionally stabilised RO_2 species. Parameter k'_{loss} is the pseudo-first order rate coefficient for the slow loss of OH radicals via diffusion and reaction with the OH precursor (H_2O_2). During the global fit k'_{loss} was fixed to the value obtained by analysing the simple exponential OH decay in the absence of MF and [MF] and [O_2] were constrained to the experimental values. The fit returned k_{RS6}, k_{regen} and k_{stab}. The total bimolecular rate coefficient for R + O_2 was then calculated: k_{R+O2} = k_{regen} + k_{stab}.

Scheme S1

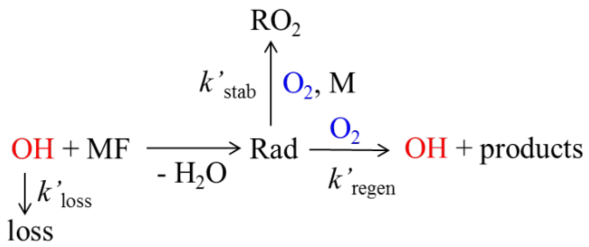

To investigate the uncertainties of the analysis using equation ES2 $k_{1,R+O2}$ was constrained to values 50% larger than $k_{1,R+O2}$ extracted by the fit to the data and then 50% lower than the extracted values. Figure S3 shows the results of the fits to the kinetic decays presented in Fig. S2 generated by constraining $k_{1,R+O2}$. The fits with $k_{1,R+O2}$ constrained does not describe well the slower component of the OH biexponential decays (insets in Figure S3), showing that the OH measurements using OH + MF/O_2 provided a reliable method for the determination of $k_{1,R+O2}$.

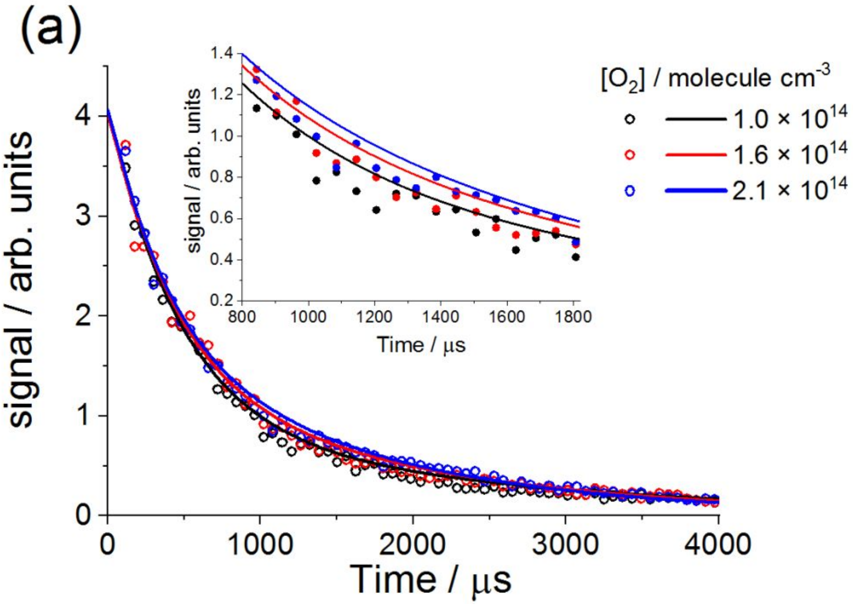

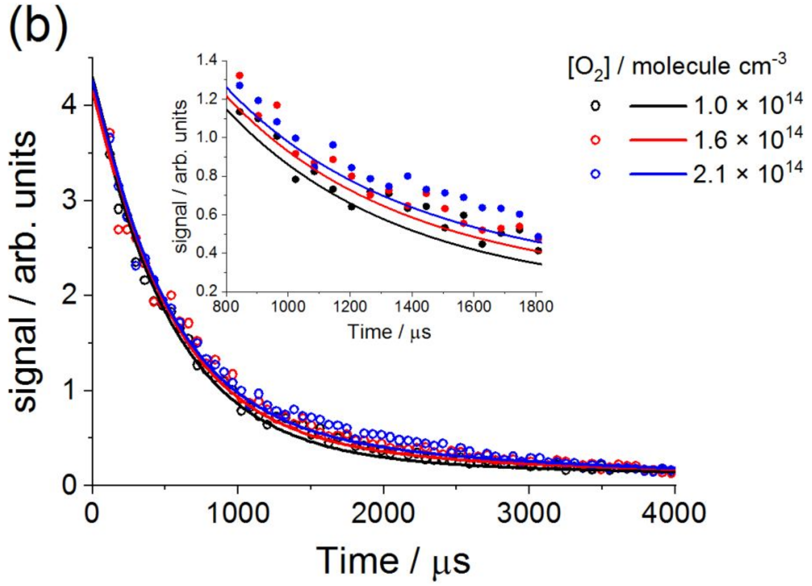

Figure S3. OH signal temporal decays collected using OH + MF/O_2 at 381 K and 6 Torr of N_2. For all traces [MF] = 4.0 × 10^{15} molecule cm^{-3}. Results of simultaneously fit using $k_{1,R+O2}$ constrained to (a) 50% larger (6.5 × 10^{-12} cm^3 $molecule^{-1}$ s^{-1}) and (b) 50% lower (2.2 × 10^{-12} cm^3 $molecule^{-1}$ s^{-1}) than $k_{1,R+O2}$ = (4.3 ± 0.8) × 10^{-12} cm^3 $molecule^{-1}$ s^{-1} retrieved by fitting equation ES2 to the observations (Fig. S2). The figure insets magnify the slower component of the decays.

S3 Kinetics of the unimolecular decomposition of CH_3OCO

$$CH_3OCO \xrightarrow{k_{2,\mathrm{dec}}} CH_3 + CO_2 \qquad \text{(R2)}$$

To characterise the unimolecular decomposition of the CH_3OCO radical (R2) theoretically, the structures of the CH_3OCO radical and its lowest energy decomposition transition state were optimised at the M062X/6-31+G level using the Gaussian code package.[1] The structure of the transition state is shown in Fig. S4. To refine the energies of the stationary points, single point energy calculations were performed at the CCSD(T)-F12/aug-CC-pVDZ level using Molpro.[2-4] The resulting zero-point energy corrected barrier height for R2 at the CCSD(T)-F12/aug-cc-pVDZ//M062X/6-311++G** level was found to be 60.3 ± ~4.0 kJ mol^{-1}.

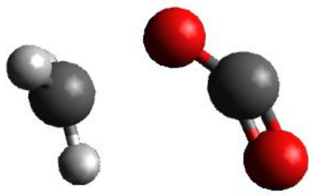

Figure S4. Structure of the decomposition transition state for the $CH_3OCO \rightarrow CH_3 + CO_2$ reaction as optimised at the M062X/6-311++G** level of theory.

A master equation analysis using the Master Equation Solver for Multi-Energy well Reactions (MESMER)[5] was used to fit the potential energy surfaces (PES) for the unimolecular decomposition of CH_3OCO to the experimental rate coefficients. The exponential down model was used to describe the collisional energy transfer with the average downward energy transferred at temperature T given by: $\langle \Delta E \rangle_{d,\,T} = \langle \Delta E \rangle_{d,\,298\,K}\,(T/298)^n$. The experimental values of $k_{2,dec}$ in the range 348–472 K at 10 Torr of Ar and 11 Torr and 50 Torr of He were fitted by constraining E_{TS} (relative to CH_3OCO) = 57.3 ± ~0.8 kJ mol^{-1}, corresponding to very high level ANL0F*”* calculations performed previously by Cho et al.,[6] whilst floating the average downward energy transferred, $\langle \Delta E \rangle_{d,\,298\,K}$ and the exponent n for each of the bath gas (Ar/He). The fit results are presented in Fig. S5 and typically agree within 85–95% with the measurements. The rigid rotor, harmonic oscillator approximation was used to calculate state densities for the majority of the modes, but all torsional degrees of freedom were treated as hindered rotors using the ‘coupledClassicalRotors’ method in MESMER. A total of two torsions were treated in the CH_3OCO and the transition state. The hindrance potentials were obtained by relaxed scans around the appropriate dihedral angles at the M062X/6-31+G** level. Tunnelling was also considered for this reaction using the WKB method built into MESMER. The 1-dimensional barrier potential was obtained from an intrinsic reaction coordinate calculation in Gaussian at the M062X/6-311++G** level of theory and corrections were applied along this potential from CCSDT-F12/aug-cc-pVTZ single point energies and projected frequency calculation to determine the change in zero-point energy. The overall potential was finally scaled to match the ANLO based energetics used in the current simulations. The calculations resulted in $\langle \Delta E \rangle_{d,\,298\,K}(Ar) = 110 \pm 30$ cm^{-1}, $n(Ar) = 0.0 \pm 0.7$ and $\langle \Delta E \rangle_{down,\,298\,K}(He) = 34.4 \pm 6.2$ cm^{-1}, $n(He) = 1.0 \pm 0.5$.

The predictions of Cho et al.[6] for $k_{2,dec}$ in the range 400–500 K and 10 Torr of He are few times larger than $k_{2,dec}$ determined from 400 K to ~500 K at 11 Torr of He in this work, even if

E_{TS} was constrained to the value found of Cho et al. in the present MESMER analysis. The authors performed high level calculations using ANL0F*"* composite method to find E_{TS} = 57.3 ± ~0.8 kJ mol^{-1}.[6] The value agrees well with the previous work (Table S1). The discrepancy between $k_{2,dec}$ calculated by this work and the predictions by Cho et al. may be due to the different values of $<\Delta E>_d$(He) used in the master equation analysis: 240 × $(T/300)^{0.85}$ cm^{-1} (MESS) and (34.4 ± 6.2) × $(T/298)^{(1.0 \pm 0.5)}$ cm^{-1} (MESMER fit to the data).

Table S1. ZPE-corrected energy of the transition state, E_{TS} for the $CH_3OCO \rightarrow CH_3 + CO_2$ reaction (reaction R1 in the main manuscript) from this work and literature

Reference	Method	E_{TS}/kJ mol^{-1}
This work	*ab initio*	60.3 ± ~4.0
Cho et al. (2023)[6]	ANL0F*"*	57.3 ± ~0.8
Tan et al. (2016)[7]	CCSD(T), MRACPF2, or MRSDCI+DS/CBS; geometries optimized at the CCSD(T)/cc-pVTZ level	56.1 – 61.9<br>59.4 on average
Tan et al. (2016)[7]	CCSD(T), MRACPF2, or MRSDCI+DS/CBS; geometries optimized at the DFT-B3LYP/ccpVTZ level	57.3 – 63.6<br>60.7 on average
Huynh et al. (2008)[8]	B3LYP/6-31G(d)	57.3
McCunn et al. (2006)[9]	CCSD(T)/aug-cc-pV(Q+d)Z//CCSD(T)/6-311G(2df,p)	61.1
McCunn et al. (2006)[9]	G3//B3LYP	57.7
Glaude et al. (2005)[10]	CBS-Q//B3LYP/6-31G(d,p)	61.5
Good and Francisco (2000)[11]	G2MP2//MP2/6-31G(d)	61.5

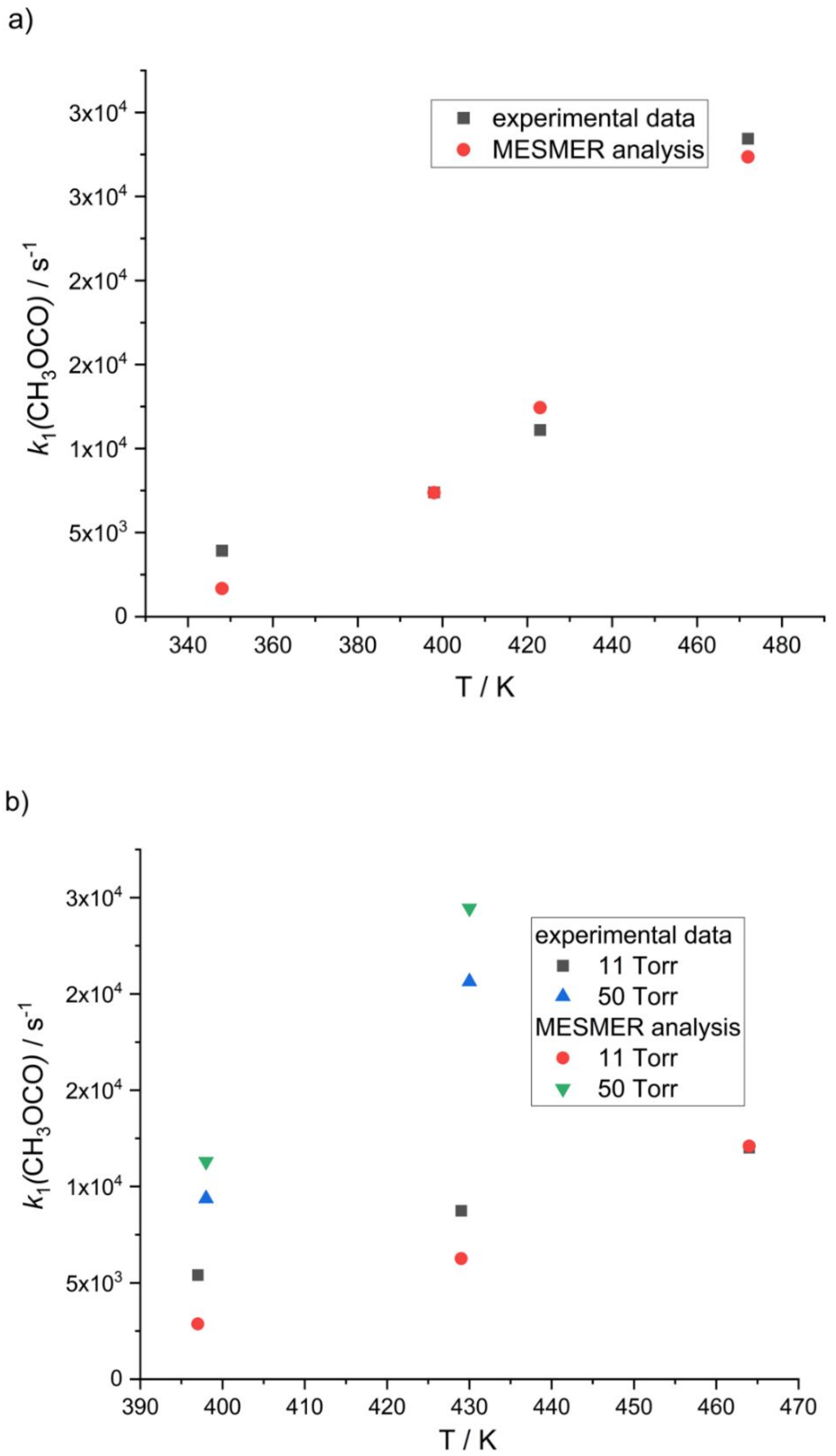

Figure S5. Temperature dependence of the rate coefficient for the CH_3OCO unimolecular decomposition studied at (a) 10 Torr of Ar – experimental data shown in black and MESMER results shown in red– and (b) 11 Torr of He – experimental data shown in black and calculated results presented in red– and 50 Torr of He –measurements shown in blue and calculations presented in green. The MESMER model uses a fixed decomposition barrier of 57.3 kJ mol^{-1} calculated by Cho et al.[6] and fitted energy transfer parameter values of $\langle \Delta E \rangle_{d,\,298\,K}$(Ar) = 110 ± 30 cm^{-1}, n(Ar) = 0.0 ± 0.7 and $\langle \Delta E \rangle_{down,\,298\,K}$(He) = 34.4 ± 6.2 cm^{-1}, n(He) = 1.0 ± 0.5.

S4 Pressure and Temperature dependence of $k_{1a,CH3OCO + O2}$ and $k_{1b,CH2OCDO + O2}$

Figure S6 shows the comparison of experimental and MESMER results with He as the bath gas. The pressure dependence of both reactions is significantly greater than the equivalent plot (Fig. 7) for Ar, where the data are closer to the high-pressure limit, consistent with more efficient energy transfer in the Ar bath gas.

These fitting results suggest that neither R + O_2 reaction has not reached its asymptotic high pressure limit and Figure S7 plots the predicted rate coefficients from the optimised MESMER models for both R+O_2 reactions as a function of total [Ar] at 213 K.

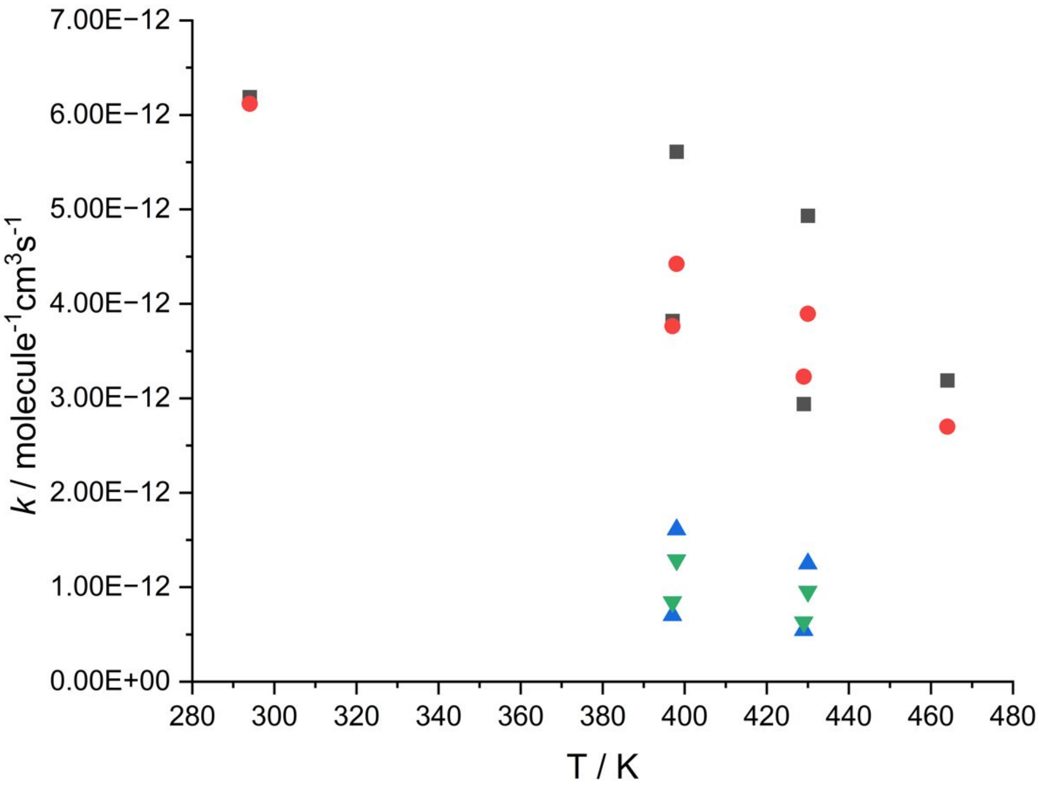

Figure S6. Comparison of experimental and MESMER rate coefficients for the CH_3OCO +O_2 (▲, Expt; ▼, MESMER fit) and the CH_2OCDO + O_2 (■, Expt; ●, MESMER fit) reactions in He.

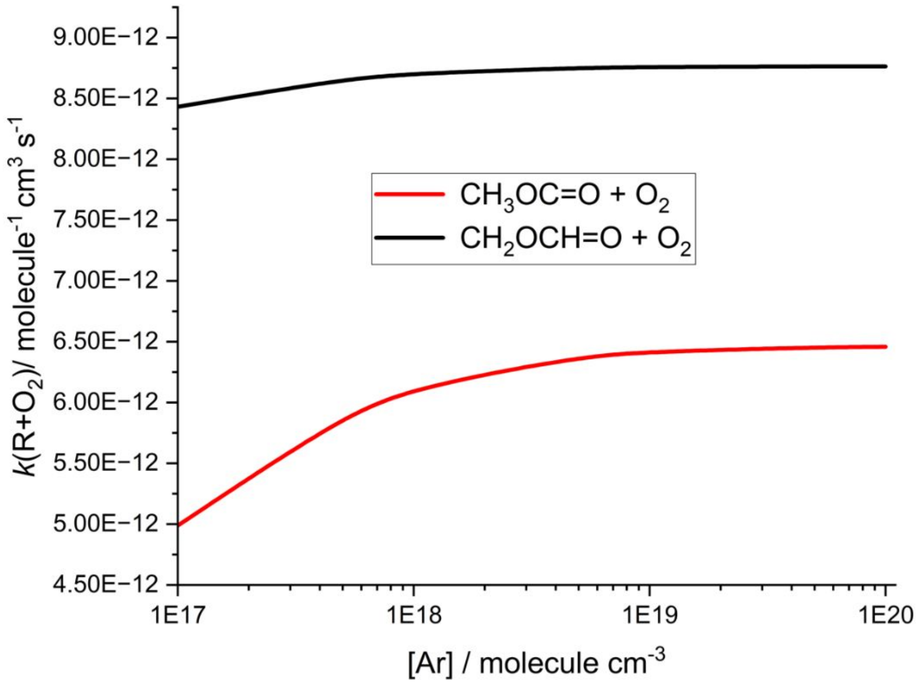

Figure S7. Calculated rate coefficients for both R + O_2 reactions at 213 K as a function of [Ar]. The x axis is plotted on a logarithmic scale.

Figure S8 shows how the ratio of $k_{1b,CH2OCDO+O2}$:$k_{1a,CH3OCO+O2}$ varies as a function of temperature. In the main text there is discussion concluding that this is due to different energy transfer parameters, so that the two reactions are at different positions in their fall-off curves.

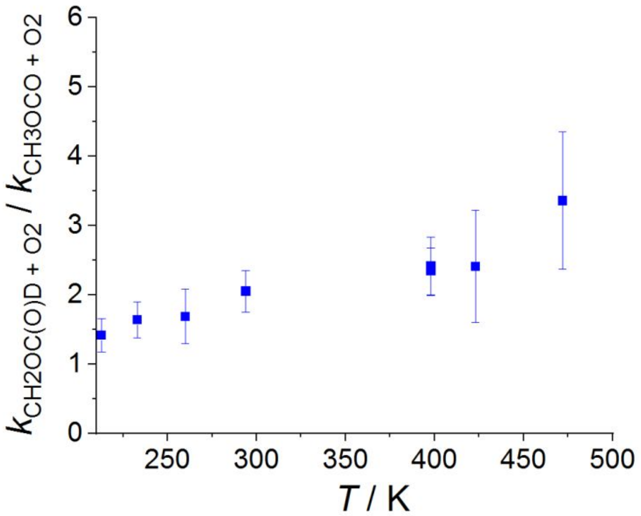

Figure S8. Plot of $k_{1b,CH2OCDO+O2}/k_{1a,CH3OCO+O2}$ against temperature

References

1. Frisch, M.; Trucks, G.; Schlegel, H.; Scuseria, G.; Robb, M.; Cheeseman, J.; Scalmani, G.; Barone, V.; Mennucci, B.; Petersson, G.; Nakatsuji, H.; Caricato, M.; Li, X.; Hratchian, H.; Izmaylov, A.; Bloino, J.; Zheng, G.; Sonnenberg, J.; Hada, M.; Fox, D., Gaussian 09 (Revision A02). *Gaussian Inc. Wallingford CT* **2009**.
2. Werner, H. J.; Knowles, P. J.; Knizia, G.; Manby, F. R.; Schütz, M., Molpro: a general-purpose quantum chemistry program package. *Wiley Interdiscip. Rev. Comput. Mol. Sci.* **2012,** *2* (2), 242-253.
3. Werner, H. J.; Knowles, P. J.; Manby, F. R.; Black, J. A.; Doll, K.; Hesselmann, A.; Kats, D.; Köhn, A.; Korona, T.; Kreplin, D. A.; Ma, Q. L.; Miller, T. F.; Mitrushchenkov, A.; Peterson, K. A.; Polyak, I.; Rauhut, G.; Sibaev, M., The Molpro quantum chemistry package. *J. Chem. Phys.* **2020,** *152* (14).
4. Werner, H. J. K., P. J.; Celani, P; Györffy, W; Hesselmann, A; Kats, D; Knizia, G.; Köhn, A; Korona, T; Kreplin, D; et. al. , MOLPRO, version , a package of ab initio programs. https://www.molpro.net.
5. Glowacki, D. R.; Liang, C. H.; Morley, C.; Pilling, M. J.; Robertson, S. H., MESMER: An Open-Source Master Equation Solver for Multi-Energy Well Reactions. *J. Phys. Chem. A* **2012,** *116* (38), 9545-9560.
6. Cho, J. Y.; Rösch, D.; Tao, Y. J.; Osborn, D. L.; Klippenstein, S. J.; Sheps, L.; Sivaramakrishnan, R., Modeling-Experiment-Theory Analysis of Reactions Initiated from Cl plus Methyl Formate. *J. Phys. Chem. A* **2023,** *127* (46), 9804-9819.
7. Tan, T.; Yang, X. L.; Ju, Y. G.; Carter, E. A., Ab Initio Reaction Kinetics of CH_3OCO and $CH_2OC(O)H$ Radicals. *Journal of Physical Chemistry B* **2016,** *120* (8), 1590-1600.
8. Huynh, L. K.; Lin, K. C.; Violi, A., Kinetic Modeling of Methyl Butanoate in Shock Tube. *J. Phys. Chem. A* **2008,** *112* (51), 13470-13480.
9. McCunn, L. R.; Lau, K. C.; Krisch, M. J.; Butler, L. J.; Tsung, J. W.; Lin, J. J., Unimolecular dissociation of the CH_3OCO radical: An intermediate in the CH_3O+CO reaction. *J. Phys. Chem. A* **2006,** *110* (4), 1625-1634.
10. Glaude, P. A.; Pitz, W. J.; Thomson, M. J., Chemical kinetic modeling of dimethyl carbonate in an opposed-flow diffusion flame. *Proc. Combust. Inst.* **2005,** *30*, 1111-1118.
11. Good, D. A.; Francisco, J. S., Tropospheric oxidation mechanism of dimethyl ether and methyl formate. *J. Phys. Chem. A* **2000,** *104* (6), 1171-1185.
